# Social Fear Memory Requires Two Stages of Protein Synthesis in Mice

**DOI:** 10.3390/ijms21155537

**Published:** 2020-08-02

**Authors:** Johannes Kornhuber, Iulia Zoicas

**Affiliations:** 1 Department of Psychiatry and Psychotherapy, Friedrich-Alexander University Erlangen-Nürnberg (FAU), 91054 Erlangen, Germany; Johannes.Kornhuber@uk-erlangen.de; 2 Department of Behavioural and Molecular Neurobiology, University of Regensburg, 93040 Regensburg, Germany

**Keywords:** social fear, social anxiety, anisomycin, protein synthesis, social investigation, fear learning, fear acquisition, fear extinction, fear memory, memory consolidation

## Abstract

It is well known that long-term consolidation of newly acquired information, including information related to social fear, require de novo protein synthesis. However, the temporal dynamics of protein synthesis during the consolidation of social fear memories is unclear. To address this question, mice received a single systemic injection with the protein synthesis inhibitor, anisomycin, at different time-points before or after social fear conditioning (SFC), and memory was assessed 24 h later. We showed that anisomycin impaired the consolidation of social fear memories in a time-point-dependent manner. Mice that received anisomycin 20 min before, immediately after, 6 h, or 8 h after SFC showed reduced expression of social fear, indicating impaired social fear memory, whereas anisomycin caused no effects when administered 4 h after SFC. These results suggest that consolidation of social fear memories requires two stages of protein synthesis: (1) an initial stage starting during or immediately after SFC, and (2) a second stage starting around 6 h after SFC and lasting for at least 5 h.

## 1. Introduction

The capability of animals to form new memories is essential for their survival and for appropriate adaptation to a complex and often-changing environment. Memory can be divided into several sequential phases, including acquisition, consolidation, retention, and retrieval. In simplistic terms, acquisition can be defined as the encoding of new information, consolidation is the stabilization of the new information for storage, retention is the preservation of the information, and retrieval is the later recall of the information [1]. During consolidation, the initial labile memory is stabilized into long-term memory, a process that requires synaptic plasticity. This process relies on different molecular mechanisms, including activation of gene transcription [2] and de-novo protein synthesis [3]. Indeed, blockade of protein synthesis using protein synthesis inhibitors was shown to attenuate the consolidation of different types of memories, including object memories [4], spatial memories [5], social memories [6,7], and cued and contextual fear memories [8,9,10,11,12,13]. It has also been reported that two distinct stages of protein synthesis are involved in memory consolidation: an initial stage starting during or immediately after training and a second stage starting approximately 3 to 6 h after training [5,6,7,8,9,10,14,15]. Interestingly, Bourtchouladze et al. [9] have shown that contextual fear memory requires either one or two stages of protein synthesis, depending on the nature of training. As such, weak training was shown to require two stages of protein synthesis, whereas stronger training was shown to require only one stage, suggesting that different training protocols may recruit common signaling pathways in distinct ways [9]. However, the requirement for protein synthesis in the consolidation of social fear memories is less clear. The protein synthesis inhibitor anisomycin failed to attenuate the consolidation of social fear memories when administered before training into the medial prefrontal cortex [16], ventral hippocampus [17], lateral septum [18], and medial amygdala [19], although these brain regions were shown to be key components of a neural circuitry mediating the formation of social fear memories in a model of conditioned defeat in Syrian hamsters [16,17,18]. However, when administered into the basolateral amygdala before training, anisomycin caused a reduction in the expression of social fear [19], suggesting that de-novo protein synthesis is required for the consolidation of social fear memories in a brain region-specific manner.

In this study, we aimed to investigate the temporal dynamics of protein synthesis during the consolidation of social fear memories. For this purpose, we administered anisomycin systemically (intraperitoneal; i.p.) at different time-points before or after social fear conditioning (SFC). Anisomycin administration (i.p.) was shown to inhibit protein synthesis in the brain for up to 6 h [20]. During SFC, mice receive mild electric foot shocks while investigating an unknown conspecific [21,22]. This training results in long-term social fear, which is expressed as reduced investigation of both known and unknown conspecifics [21]. The expression of SFC-induced social fear relies on the dorsal hippocampus, central amygdala, and dorsolateral septum [23,24], although other brain regions might also be involved. As only a short training session is needed to induce robust and long-lasting social fear memory, this paradigm is ideal for examining the molecular mechanisms contributing to the different memory stages.

## 2. Results

### Anisomycin Impairs the Consolidation of Social Fear Memory in a Time-Point-Dependent Manner

**Figure 1:**
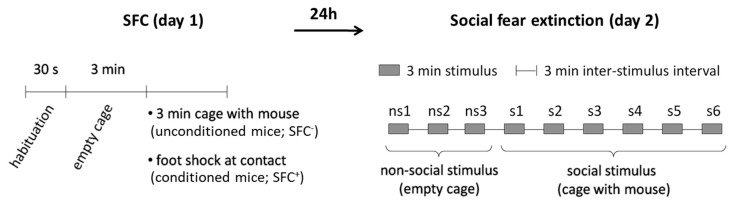
Schematic representation of the social fear conditioning (SFC) paradigm. During SFC on day 1, mice were placed in the conditioning chamber and, after a 30-s habituation period, an empty wire mesh cage was placed inside as a non-social stimulus. After 3 min, the empty cage was replaced by an identical cage containing an unfamiliar mouse. Unconditioned mice (SFC^−^) were allowed to investigate this social stimulus for 3 min. Conditioned mice (SFC^+^) were given a mild electric foot shock each time they investigated the social stimulus. During social fear extinction on day 2, mice were exposed in their home cages to three non-social stimuli, followed by exposure to six unfamiliar social stimuli. Anisomycin or vehicle were administered intraperitoneally either 20 min before, immediately after, 4 h, 6 h, or 8 h after SFC on day 1.

**Figure 2:**
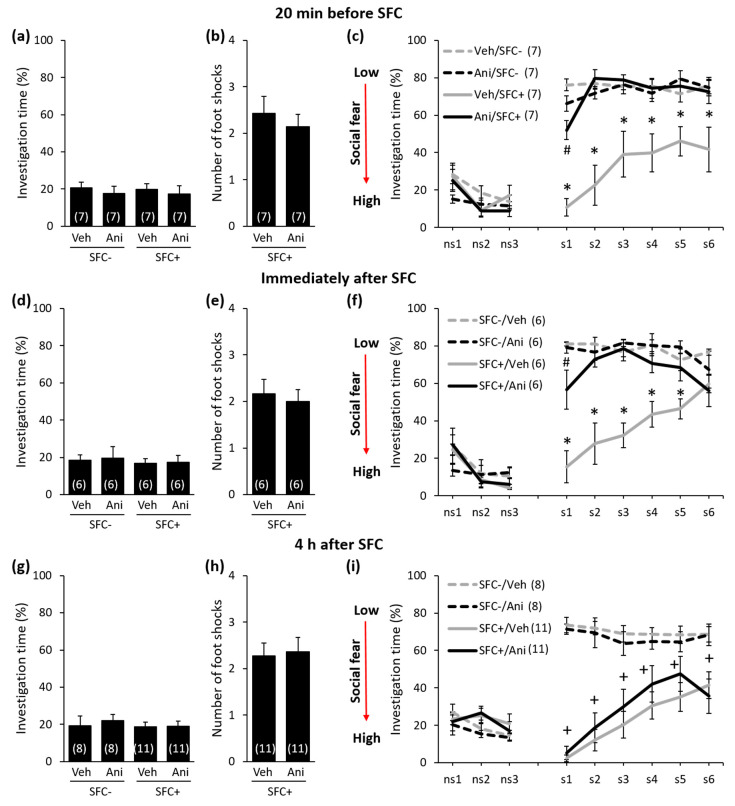
Anisomycin impairs the consolidation of social fear memory when administered 20 min before or immediately after social fear conditioning (SFC), but not when administered 4 h after SFC. (**a**,**d**,**g**) Pre-conditioning investigation of the non-social stimulus (empty cage) shown by unconditioned and conditioned mice (SFC^−^ and SFC^+^, respectively) during SFC on day 1. (**b**,**e**,**h**) Number of foot shocks received by SFC^+^ mice during SFC. (**c**,**f**,**i**) Investigation of the non-social (ns1–ns3) and social (cages with mice; s1–s6) stimuli during social fear extinction on day 2. Mice were injected intraperitoneally either with vehicle (Veh; 0.9% saline; 5 mL/kg) or anisomycin (Ani; 150 mg/kg) at different time-points before or after SFC. Data represent mean ± SEM and numbers in parenthesis indicate group sizes. *p* < 0.05 * versus all groups; # versus Veh/SFC^+^ and Ani/SFC^−^; + versus respective SFC^−^ controls.

**Figure 3:**
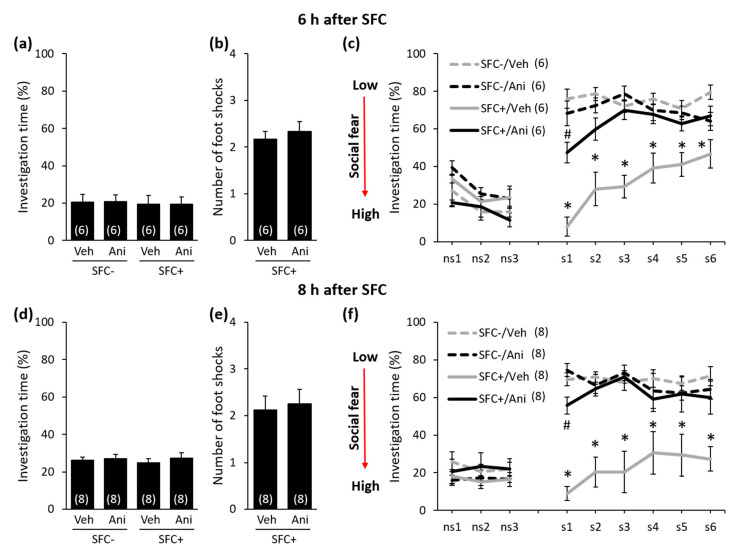
Anisomycin impairs the consolidation of social fear memory when administered 6 h or 8 h after social fear conditioning (SFC). (**a**,**d**) Pre-conditioning investigation of the non-social stimulus (empty cage) shown by unconditioned and conditioned (SFC^−^ and SFC^+^, respectively) mice during SFC on day 1. (**b**,**e**) Number of foot shocks received by SFC^+^ mice during SFC. (**c**,**f**) Investigation of the non-social (ns1–ns3) and social (cages with mice; s1–s6) stimuli during social fear extinction on day 2. Mice were injected intraperitoneally either with vehicle (Veh; 0.9% saline; 5 mL/kg) or anisomycin (Ani; 150 mg/kg) 6 h or 8 h after SFC. Data represent mean ± SEM and numbers in parenthesis indicate group sizes. *p* < 0.05 * versus all groups; # versus SFC^+^/Veh and SFC^−^/Ani.

In order to investigate the temporal dynamics of protein synthesis during the consolidation of social fear memories, we studied conditioned mice (SFC^+^ mice) and unconditioned control mice (SFC^−^ mice) that received a single i.p. injection either with anisomycin or vehicle (0.9% saline) either 20 min before, immediately after, 4 h, 6 h, or 8 h after training in the SFC paradigm (day 1; Figure 1, Figure 2 and Figure 3). SFC^+^ mice received mild electric foot shocks each time they investigated an unknown conspecific, whereas SFC^−^ mice investigated an unknown conspecific without receiving foot shocks. Twenty-four hours after SFC, during social fear extinction, we assessed the time that the SFC^+^ and SFC^−^ mice spent investigating six unknown conspecifics (i.e., social investigation) as a read-out of social fear memory (Figure 1).

During SFC, all mice spent a similar amount of time investigating the non-social stimulus (empty cage), which reflects similar pre-conditioning non-social anxiety levels between the groups (Figure 2a: F(3,24) = 0.208; *p* = 0.890; Figure 2d: F(3,20) = 0.091; *p* = 0.964; Figure 2g: F(3,34) = 0.205; *p* = 0.893; Figure 3a: F(3,20) = 0.038; *p* = 0.990; Figure 3d: F(3,28) = 0,248; *p* = 0.862). Furthermore, both anisomycin- and vehicle-treated SFC^+^ mice received a similar number of foot shocks during SFC, which indicates that all SFC^+^ mice experienced similar levels of distress during SFC. This also indicates that anisomycin did not impair the acquisition/learning of social fear (Figure 2b: T(12) = 0.632; *p* = 0.539; Figure 2e: T(10) = 0.415; *p* = 0.687; Figure 2h: T(20) = −0.220; *p* = 0.828; Figure 3b: T(10) = 0.549; *p* = 0.549; Figure 3e: T(14) = −0.290; *p* = 0.776). Twenty-four hours later, during social fear extinction, all SFC^+^ and SFC^-^ mice showed similar investigation of the non-social stimuli (three empty cages; ns1–ns3), which indicates that SFC did not induce unspecific non-social fear (Figure 2c,f,i and Figure 3c,f). However, all vehicle-treated SFC^+^ mice spent less time investigating the social stimuli (six unknown conspecifics; s1–-s6) compared with vehicle-treated SFC^-^ mice, which indicates intact social fear memory. Anisomycin, on the other hand, caused a reduction in the expression of social fear when administered 20 min before SFC (Figure 2c; conditioning × treatment effect F(1,24) = 22.968; *p* < 0.001; stimulus × conditioning × treatment effect F(8,192) = 4.921; *p* < 0.001), immediately after SFC (Figure 2f; conditioning × treatment effect F(1,20) = 17.086; *p* = 0.001; stimulus × conditioning × treatment effect F(8,160) = 3.277; *p* = 0.002), 6 h after SFC (Figure 3c; conditioning × treatment effect F(1,20) = 19.523; *p* < 0.001; stimulus × conditioning × treatment effect F(8,160) = 7.815; *p* < 0.001), and 8 h after SFC (Figure 3f; conditioning × treatment effect F(1,28) = 24.828; *p* < 0.001; stimulus × conditioning × treatment effect F(8,224) = 2.073; *p* = 0.039), but not when administered 4 h after SFC (Figure 2i; conditioning × treatment effect F(1,34) = 0.698; *p* = 0.409; stimulus × conditioning × treatment effect F(8,272) = 0.639; *p* = 0.744). This finding indicates that anisomycin impaired the consolidation of social fear memories at all time-points, except when administered 4 h after SFC.

## 3. Discussion

Our results show for the first time that social fear memory requires two stages of protein synthesis in male mice: (1) an initial stage starting during or immediately after SFC (i.e., acquisition of social fear), and (2) a second stage starting around 6 h after SFC. These findings extend previous reports showing that the consolidation of both aversive and non-aversive memory is dependent on at least two stages of protein synthesis [5,6,7,8,9,10,14,15]. The initial stage of protein synthesis was reported to last up to 1.5 h [5,9,14,15], whereas the temporal dynamics of the second stage seem to depend on the type of memory. For example, the second stage of protein synthesis required for the consolidation of object memory was shown to occur between 3 h and 6 h after training [4], for social memory between 6 h and 18 h after training [6,7], for spatial memory between 4 h and 6 h after training [5], for contextual fear memory between 4 h and 11.5 h after training [13], for cued fear memory between 4 h and 8 h after training [9], and for inhibitory avoidance memory between 3 h and 7.5 h after training [8,10,14,15]. Given that the concentration of anisomycin used in this study inhibits most of the protein synthesis for 2 to 3 h [3,25], we showed that the second stage of protein synthesis required for the consolidation of social fear memory occurred between 6 h and at least 11 h after training. Interestingly, even motor memories were shown to require two stages of protein synthesis: the initial stage occurring within 30 min after training and the second stage occurring between 6 h and at least 14 h after training for rotarod running [26].

Independent of the temporal dynamics of protein synthesis, the initial stage seems to involve the expression of transcription factors and immediate early genes. The second stage might involve the expression of structural genes whose protein products are required for long-lasting synaptic remodeling necessary for the formation of long-term memories [15,27,28]. For example, consolidation of social memory was shown to coincide with an increase in c-Fos expression during the initial, but not during the second stage of protein synthesis, in brain areas involved in the processing of olfactory cues, such as the main olfactory bulb, piriform cortex, medial preoptic area, and medial amygdala [6]. Between the two stages of protein synthesis, however, the consolidation of social memory was insensitive to systemically administered anisomycin and coincided with the decline in the number of c-Fos-positive neurons [6]. It is well known that the activity of signaling pathways that involve mitogen-activated protein kinase (MAPK), cyclic adenosine monophosphate (cAMP), and protein kinase A (PKA) play a critical role in neuronal plasticity that underlie the hippocampus- and amygdala-dependent learning and memory processes [28]. Therefore, it is not surprising that the second stage of protein synthesis was reported to coincide with an increase in PKA activity [9], cAMP levels, and phospho-cAMP response element-binding protein (CREB) immunoreactivity [29], and an activation of the ERK1/2 and CREB pathway [30]. As such, the PKA inhibitor Rp-cAMP was shown to impair the consolidation of contextual fear memories when administered intracerebroventricularly before each stage of protein synthesis [9]. A similar impairing effect on inhibitory avoidance memories was reported after administration of the PKA inhibitor KT5720 into the hippocampus [29]. Furthermore, intra-hippocampal treatment with the MEK inhibitor U0126 before each stage of protein synthesis prevented the activation of both ERK1/2 and CREB and thereby impaired the consolidation of cued fear memories [30]. Given that anisomycin can modulate intracellular signaling cascades independently of its effect on protein synthesis [31], it is unclear how and whether anisomycin impairs consolidation of social fear memory by modulating these signaling pathways. The dose of anisomycin might play an important role, as only higher doses of anisomycin inhibit protein synthesis [32,33], whereas lower doses are sufficient to induce c-Fos expression and activation of MAPK [31,34]. Independent of the exact mechanism, our results, together with these studies, suggest that the function of the second stage of protein synthesis depends on the integrity of the first stage.

Impaired memories after pre-training administration of protein synthesis inhibitors might also reflect confining effects on learning processes and thus on memory acquisition. In our study, however, the impaired social fear memory observed after anisomycin administration 20 min before SFC was unlikely to be due to a direct effect on the acquisition of social fear. As such, both vehicle- and anisomycin-treated SFC^+^ mice received a similar number of foot shocks during SFC, indicating similar social fear learning. In support of a lack of involvement of protein synthesis in the acquisition of conditioned fear, Bourtchouladze et al. [9] reported that intracerebroventricular administration of anisomycin 3 h before training, a time-point at which the acquisition, but not the consolidation would be affected, did not influence the expression of contextual fear memories 24 h later. However, whether protein synthesis is required for the acquisition of social fear memory remains to be investigated in future studies implying administration of anisomycin 3 h before SFC.

Given that anisomycin was systemically administered in our study, we can only speculate on the brain regions in which protein synthesis is necessary for the consolidation of social fear memories. As the dorsal hippocampus, central amygdala, and dorsolateral septum play an essential role in the consolidation and expression of SFC-induced social fear [23,24], protein synthesis might occur within one or more of these brain regions. The dependency of aversive social memories on protein synthesis within specific amygdala nuclei has been already suggested in a model of conditioned defeat in Syrian hamsters [19]. As such, post-training administration of anisomycin into the basolateral amygdala led to a reduction in the expression of conditioned fear, whereas administration into the medial amygdala caused no effects [19].

Taken together, our results add a novel piece of information to the temporal dynamics of protein synthesis during the consolidation of aversive memories, and suggest that social fear memories require at least two stages of protein synthesis: (1) an initial stage starting immediately after social fear learning, and (2) a second stage starting 6 h after learning and lasting for at least 5 h.

## 4. Materials and Methods

### 4.1. Animals

Male CD1 mice (Charles River, Sulzfeld, Germany, 10 weeks of age) were individually housed for one week before the experiments started and remained single-housed throughout the experiments, to allow for maintenance of social fear in SFC^+^ mice. Mice were kept under standard laboratory conditions (12:12 light/dark cycle, lights on at 07:00 h, 22 °C, 60% humidity, food and water ad libitum). Experiments were performed during the light phase between 09:00 and 14:00 in accordance with the Guide for the Care and Use of Laboratory Animals of the Government of Unterfranken and the guidelines of the NIH. All efforts were made to minimize animal suffering and to reduce the number of animals used. 

### 4.2. Social Fear Conditioning (SFC) Paradigm

To induce social fear, mice were conditioned during SFC and social investigation was assessed 24 h later during social fear extinction as a read-out of social fear memory.

*SFC.* SFC was performed with a computerized fear conditioning system (TSE System GmbH, Bad Homburg, Germany) as previously described [21,22,23,24,35,36,37,38]. Mice were placed in the conditioning chamber (45 × 22 × 40 cm) and, after a 30-s habituation period, an empty wire mesh cage (7 × 7 × 6 cm) was placed as a non-social stimulus near one of the short walls. After 3 min, the non-social stimulus was replaced by an identical cage containing an unfamiliar mouse. Unconditioned mice (SFC^−^) were allowed to investigate this social stimulus for 3 min, whereas conditioned mice (SFC^+^) were given a 1-sec mild electric foot shock (0.7 mA) each time they investigated, i.e., made direct contact with the social stimulus. Mice received between one and four foot shocks with a variable inter-shock interval, depending on when direct social contact was made. The number of foot shocks was assessed as a measure of distress and of social fear learning/acquisition. Mice were returned to their home cage when no further social contact was made for 2 min (average duration of SFC approximately 10 min). The time that the mice spent investigating the non-social stimulus as a pre-conditioning measure of non-social anxiety was analyzed.

*Social fear extinction.* Twenty-four hours after SFC, mice were exposed in their home cages to three non-social stimuli, i.e., empty cages identical to the cage used during SFC, to assess non-social investigation as a parameter of non-social fear. Mice were then exposed to six unfamiliar social stimuli, i.e., mice enclosed in wire mesh cages, to assess social investigation as a parameter of social fear. Each stimulus was placed near a short wall of the home cage and presented for 3 min with a 3-min inter-exposure interval. The test was recorded and analyzed using JWatcher (V 1.0, Macquarie University, Sydney, Australia and UCLA, Los Angeles, CA, USA). Non-social investigation was defined as direct sniffing of the empty cage, whereas social investigation was defined as direct sniffing of the cage and/or of the social stimulus inside of the cage.

### 4.3. Blockade of Protein Synthesis

Anisomycin (Sigma–Aldrich, Darmstadt, Germany) was diluted in 0.9% saline by adding 0.1 M HCl, after which the pH was adjusted to 7.2 using 0.1 M NaOH. Mice were injected i.p. either with anisomycin (150 mg/kg) at a volume of 5 mL/kg or with an equivalent volume of vehicle solution (0.9% saline) either 20 min before, immediately after, 4 h, 6 h, or 8 h after SFC. At the concentration used, anisomycin was shown to inhibit more than 96% of protein synthesis for 2 to 3 h [3,25].

### 4.4. Statistical Analysis

For statistical analysis, SPSS (Version 21, SPSS Inc., Chicago, IL, USA) was used. Data were analyzed using the Student’s t-test, one- or three-way analysis of variance (ANOVA) for repeated measures, followed by Bonferroni’s post-hoc analysis whenever appropriate. Statistical significance was set at *p* < 0.05.
